# Transgenerational Effects of Heavy Metal Pollution on Immune Defense of the Blow Fly *Protophormia terraenovae*

**DOI:** 10.1371/journal.pone.0038832

**Published:** 2012-06-12

**Authors:** Mari Pölkki, Katariina Kangassalo, Markus J. Rantala

**Affiliations:** Department of Biology, Section of Ecology, University of Turku, Turku, Finland; Glasgow Caledonian University, United Kingdom

## Abstract

Recently environmental conditions during early parental development have been found to have transgenerational effects on immunity and other condition-dependent traits. However, potential transgenerational effects of heavy metal pollution have not previously been studied. Here we show that direct exposure to heavy metal (copper) upregulates the immune system of the blow fly, *Protophormia terraenovae,* reared in copper contaminated food. In the second experiment, to test transgenerational effects of heavy metal, the parental generation of the *P. terraenovae* was reared in food supplemented with copper, and the immunocompetence of their offspring, reared on uncontaminated food, was measured. Copper concentration used in this study was, in the preliminary test, found to have no effect on mortality of the flies. Immunity was tested on the imago stage by measuring encapsulation response against an artificial antigen, nylon monofilament. We found that exposure to copper during the parental development stages through the larval diet resulted in immune responses that were still apparent in the next generation that was not exposed to the heavy metal. We found that individuals reared on copper-contaminated food developed more slowly compared with those reared on uncontaminated food. The treatment groups did not differ in their dry body mass. However, parental exposure to copper did not have an effect on the development time or body mass of their offspring. Our study suggests that heavy metal pollution has positive feedback effect on encapsulation response through generations which multiplies the harmful effects of heavy metal pollution in following generations.

## Introduction

Heavy metals are known to transfer from soil to plants and from plants to herbivores. In polluted areas such as in the vicinity of metal industry, heavy metal pollution has been found to accumulate in plants and various invertebrates [1]–[3]. Several studies have documented the disadvantageous effects of heavy metal pollution on different traits. Individuals exposed to heavy metals achieve smaller size [4], have longer development time [5] and are shown to be more sensitive to various parasites and pathogens [6], [7]. In addition, high concentrations of heavy metals are known to have an effect on survival [8], reproductive success [8], longevity and fecundity [5]. Long term exposure is thought to lead to populations of metal-tolerant phenotypes [9], [10], which might explain why individuals gathered from polluted areas are found to be more successful in heavy metal containing conditions compared with individuals from unpolluted areas [11]. Even though the direct adverse effects of heavy metal pollution on invertebrates are quite well understood, studies testing transgenerational effects of parental exposure to heavy metal pollution on offspring immunity are so far lacking.

Environmental conditions experienced by parents have been found to have an effect on offspring phenotype brought about by the transmission of nongenetic factors instead of genetic inheritance to offspring [12]–[14]. Previously, empirical studies considering parental effects have demonstrated that environmental conditions during parental development impact on life-history traits of their offspring [15]–[17]. Sadd et al. [18] have found evidence of transgenerational immune priming in the bumblebee, *Bombus terrestris* L. Those queens given an immune-challenge treatment prior to the colony founding were producing workers with higher levels of antibacterial activity [18]. There is increasing empirical support that environmental conditions experienced by parents can produce epigenetic adjustments [19], [20]. The expression of immune functions through non-heritable effects that regulate the genome activity such as DNA methylation, histone modifications, chromatin structure and noncoding RNA, are considered as epigenetic factors [21]. Previously, environmentally induced transgenerational epigenetic changes have been demonstrated in rats [22], [23] and in mice [24]. Whereas in vertebrates transgenerational effects have received notably more interest, experimental studies of these effects in invertebrates are poorly understood.

Unlike in vertebrates, invertebrates lack acquired immunity, showing far less complicated immune defense systems, even though several components of their immune systems are homologous [25]. Therefore, insects are an excellent model organism for studies considering the effects of environmental pollution on the immune system. Immunity of insects against pathogens is based on the innate immune system that consists of humoral and cellular responses [26]. The humoral immune system produces a variety of circulating antibacterial and antifungal peptide (humoral factors) secretions which are used against microbial pathogens as a second line defense [26], [27] whereas cell-mediated immune responses such as nodulation, phagocytosis and encapsulation are primarily used against multicellular intruders [26], [28]. Of these, encapsulation is the main defense mechanism against multicellular organisms [26] and viruses [29]. A foreign intruder(s) is eliminated by capsulating it with hematocytes [26], [30]. Previous study on the geometrid moth, *Epirrita autumnata,* has found that exposure to heavy metals, copper and nickel, have an effect on immunity when measured as an encapsulation response against an artificial antigen – a nylon monofilament. Moderate amounts (used amounts were derived from the maximum found naturally from birch leaves) were found to enhance immunity whereas higher concentrations debased it [31], indicating that heavy metals might have direct disadvantageous effects on insect immunity. Of heavy metals, copper (Cu) is an important trace element essential for the maintenance of numerous metabolic processes, and it is more easily regulated in body tissues than non-essential metals, such as cadmium, mercury and lead [32]. To our knowledge, this is the first study to test whether changes in immunity will remain in population even if the environmental conditions have returned to normal and the heavy metal load is no longer apparent.

The aim of the present experiment was to study whether direct exposure to heavy metal had an effect on immunity and more particularly, whether parental exposure to heavy metal pollution through larval diet had any transgenerational effects on offspring immunity by using the blow fly *Protophormia terraenovae* (Diptera: Calliphoridae; Robineau-Desvoidy, 1830) as a model species. To investigate the direct effects of heavy metal exposure to parental innate immune response, maggots of the parental generation were reared in food supplemented with copper. Then to test transgenerational effects, the next generation was bred in uncontaminated food. Immunity was tested by measuring the encapsulation response against an artificial antigen (nylon monofilament) from adult flies of both generations. Here we show first that exposure to copper, activates the immunity of *P. terraenovae* even in low concentrations and then we demonstrate that parental larval exposure to an environmental pollutant has transgenerational effects on immunity.

## Results

### Experiment 1

**Figure 1:**
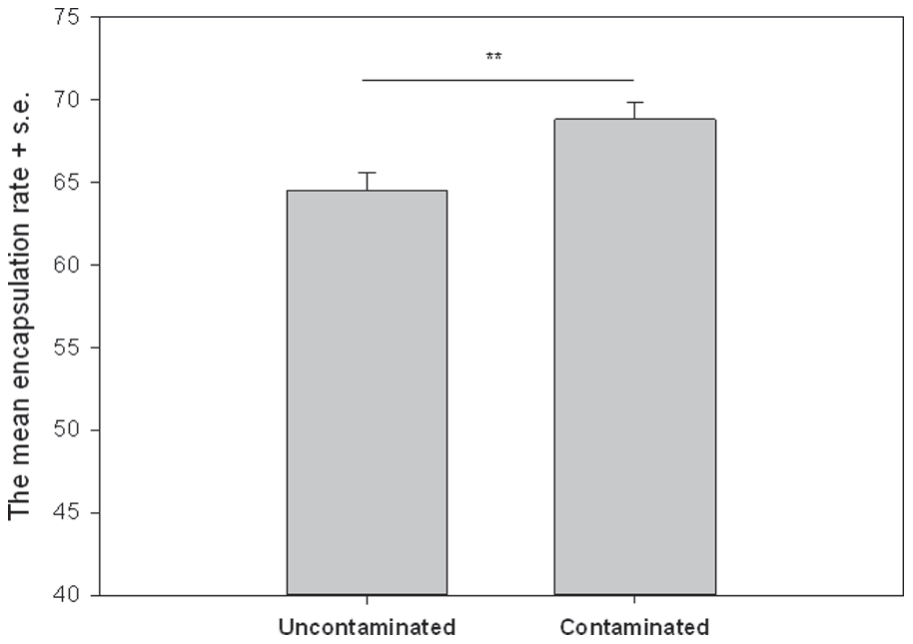
The mean encapsulation rate (artificial unit) of the parental generation reared in copper contaminated and uncontaminated environments (uncontaminated: N=189, mean =64.53, SD =14.54; contaminated: N=184, mean =68.82, SD =14.24). The encapsulation rate was measured as average gray value of reflected light, which is considered as relative darkness (for more details see Materials and methods).

Direct exposure to copper had an effect on innate immunity (t test, t=−2.876, df=371, *P*=0.004). Those individuals that were reared in copper contaminated food had a stronger encapsulation response compared with the uncontaminated group (Fig. 1). There were no differences between sexes (ANOVA: F_1,369=_0.250, *P*=0.705) and there was no interaction between treatment and sex (ANOVA: F_1,369=_0.846, *P*=0.358) or between treatment and vials (nested ANOVA: F_10,361=_1.510, *P*=0.134) in encapsulation response. Heavy metal treatment did not have any effect on the survival of the flies (ANOVA: F_1,11=_1.106, *P*=0.318).

However, direct exposure to copper did not have any effect on the mean dry body mass (t test, t=1.102, df=15, *P*=0.287) and there were no differences in mean body mass between females and males (t test, uncontaminated: t=−0.228, df=10, *P*=0.825; contaminated: t=1.984, df=10, *P*=0.075). Instead, exposure to copper had an effect on development time (Mann-Whitney U test, U=3879.0, Z=−14.923, *P*=0.0001, n=200 uncontaminated/ 197 contaminated). In the copper contaminated group, individuals developed substantially slower (n=197, mean 16.50, SD=0.46) than in the uncontaminated group (n=200, mean 15.19, SD=0.40). However, there were no differences between sexes (Mann-Whitney U test, uncontaminated: U=4741.5, Z=−0.766, *P*=0.444, n=96 females/ 104 males; contaminated: U=4648.5, Z=−0.629, *P*=0.529, n=91 females/ 106 males) in development time.

### Experiment 2

**Figure 2:**
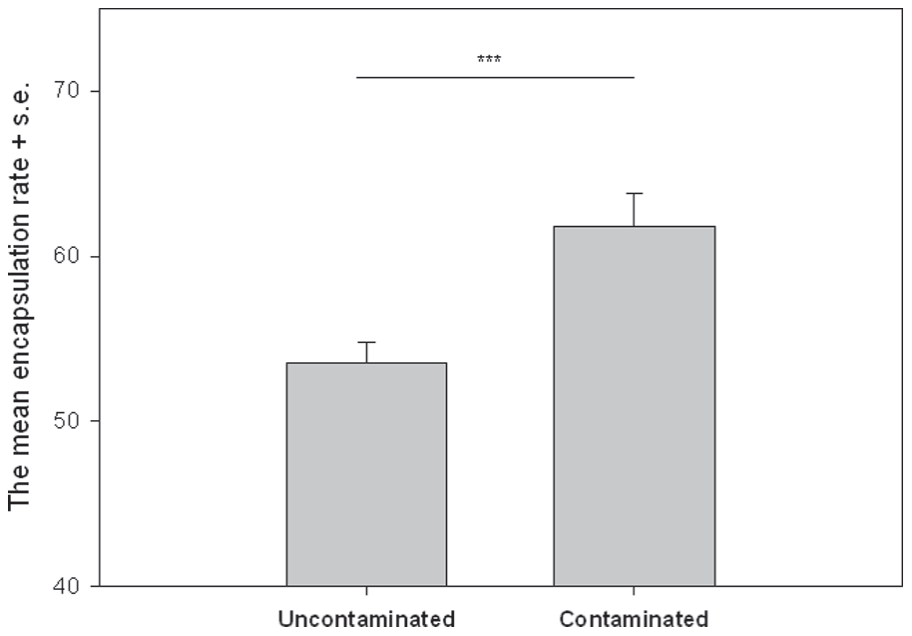
The mean encapsulation response of the offspring whose parents were reared in copper contaminated and uncontaminated environments (uncontaminated: N=90, mean =53.52, SD =11.97; contaminated: N=92, mean =61.78, SD =19.29).

Parental exposure to heavy metal pollution had an effect on their offspring's immunity (t test, t=−3.463, df=164, *P*=0.001). Individuals whose parents were reared in copper contaminated food had a stronger encapsulation response compared with the uncontaminated group (Fig. 2). The sexes did not differ in encapsulation response (ANOVA: F_1,178=_6.846, *P*=0.232). There was no interaction in encapsulation response between sexes and treatment groups (ANOVA: F_1,178=_0.062, *P*=0.804) and encapsulation response did not differ between individuals of different vials (nested ANOVA: F_11,169=_1.130, *P*=0.341).

In our study, direct parental exposure to copper did not have any effect on the dry body mass of the next generation (t test, t=1.072, df=21, *P*=0.296). There was no difference between sexes in the dry body mass (t test, uncontaminated: t=0.799, df=10, *P*=0.443; contaminated: t=1.221, df=9, *P*=0.253). In addition, parental exposure to copper did not have an effect on the length of the development time of their offspring (Mann-Whitney U test, U=5289.0, Z=−0.501, *P*=0.616, n=97 uncontaminated/ 113 contaminated). There were no differences between sexes in the length of the development time (Mann-Whitney U test, uncontaminated: U=1098.5, Z=−0.651, *P*=0.515, n=50 females/ 47 males; contaminated: U=1497.5, Z=−0.243, *P*=0.808, n=59 females/ 52 males).

## Discussion

Here, we show that exposure to copper during early parental development stages has an effect on offspring innate immunity in the blow fly, *P. terraenovae* (Diptera). Furthermore, the changes in immunity will remain in the population even if the environmental conditions have returned to normal and the heavy metal load is no longer apparent. Our results are consistent with the previous studies on the geometrid moth, *Epirrita autumnata* (Lepidoptera) [31], [33] and wild ant colonies of *Formica aquilonia* (Hymenoptera) [7], in which exposure to copper was found to strengthen the immunity at low to medium levels. Furthermore, in the recent study of Dubovskiy et al. [6] a low concentration of nickel was found to increase glutathione S-transferase (GST), phenoloxidase activity and encapsulation response levels. Previous findings on a variety of Mollusca species indicate that the intensifying effect of copper on immune response might result from an increase in the number of hemocytes [34] or granulocytes [35], [36]. Recently, encapsulation response has been found to be related with the number of hemocytes in hemolyph [37]. However, insects have two kinds of immunity–cellular encapsulation and melanotic encapsulation–of which the first is more delineated in the order of Lepidoptera whereas the other is more common in Diptera [26]. In particular, hemocytes play an important role in immune reactions and are required in the encapsulation process [26], [38], where the pathogen is capsulated with several layers of hemocytes and as a consequence an intruder is destroyed.

We found that exposure to copper during early parental development stages had a transgenerational epigenetic effect on offspring immunity. Copper concentration used here had no effect on mortality of the flies, so there was no selection toward higher copper tolerance in the parental generation. To our knowledge this is the very first study to show that upregulation of immune system caused by exposure to heavy metal pollution during early parental development stages will remain in the next generation. Our finding is consistent with the previous studies where parental environmental conditions were found to have a transgenerational effect on offspring life-history traits through non-genetic inheritance [16]. Environmental conditions experienced by parents, especially by the mother, are thought to play a crucial role in shaping offspring phenotype [39]. In certain conditions individuals may benefit by having upregulated immunity, particularly when parents and their offspring confront the same pathogen pressure [18]. However, upregulation of immunity in this case was a result of copper exposure and does not correspond to the pathogen load. Instead, maintaining high level immunity consumes energy and upregulation of immunity may result, for instance, in reduced reproductive success or longevity [40]. The present study is the first to show that heavy metal pollution has a transgenerational effect on innate immunity. Thus far, there are no studies to test whether the changes in immunity will remain in populations. Since invertebrates and vertebrates share homologous components in their innate immune system [25], it would be interesting to know whether the same phenomenon occurs in vertebrates.

Here, exposure to copper during early parental development stages seemed to have no effect on parental or offspring adult size. The observed slower development time in the copper contaminated group (parental generation) that was fed copper supplemented food suggests resource allocation between growth rate and immunity. Maintenance of the immune responses is considered energetically costly [15], [41] and previous studies have found a trade-off between immune function, size and development time [42]. This indicates that perceived difference in development times of parental generation is more likely a result of toxic effects of copper. The detoxification process of harmful compounds consumes energy, which is why less energy remains for growth [43]. In an environment containing toxic compounds it might take longer to achieve a certain size, as resources are needed for regulating harmful compounds received from larval nourishment.

We found no difference between offspring development times of the treatment groups despite the observed difference in immune defence, suggesting that differences in immune function in offspring might be a result of transgenerational epigenetic changes caused by parental exposure to copper. Whereas, observed difference in parental development times might be a result of the regulation of toxic compounds, this might explain why the same effect does not occur in the next generation whose larval food did not contain any added copper.

Recent studies have demonstrated the deleterious effects of high heavy metal concentrations on immune defence [6], [31]. Concentration used in the present experiment was relatively low. In a preliminary test it was found to have an effect on immunity and yet to have no significant effects on mortality of the maggots or adult flies. Increase in mortality might have an effect on parental population by causing selection [18], because remaining individuals might consist of those that are able to survive in copper contaminated environment [11].

To conclude, we found that exposure to copper, during early parental development has a transgenerational effect on encapsulation response against an artificial antigen in adult flies of the blow fly, *P. terraenovae*, even in relatively low concentration. The changes in immunity will remain in the next generation even if exposure to an environmental pollutant is no longer apparent. Transgenerational epigenetic effects of environmental pollutants on invertebrate immunity and other life history traits are poorly understood. In particular, the long-lasting effects are barely known of and should definitely be studied more in future experiments. The results of our study suggest that the effect of an environmental toxin in populations might be more complicated than previously thought. This is something which should be considered when evaluating the effects of the pollutants on the viability of animal populations.

## Materials and Methods

### Study species and maintenance of the stock population

*P. terraenovae* is a large, metallic blue Calliphorid species that prefers cool temperatures, having a holarctic distribution. Maggots of this species usually nourish on carcasses and decaying meat whereas adult flies feed on nectar and fluids of decaying meat [44].

Flies for the experiment were obtained from a laboratory stock that were collected (over 600 adult flies) from Turku, South-Western Finland during the summer of 2009 and maintained at the University of Turku. Stock consists of several large cages with 200 to 300 individuals in each. Stock was maintained at a constant temperature of 24±1°C under constant light, which is the same light condition that wild flies have in Finland at midsummer. Adult flies were fed with dry powder food (consist of 1∶1∶1 sugar, baby's milk formula and semolina, mixture contains 10% of dry yeast) and fresh water *ad libitum*. Maggots were reared in cat food (Pirkka beef pâté).

### Experiment 1

In order to study whether direct exposure to heavy metal, copper, during early developmental stages has an effect on innate immune defence in *P. terraenovae*, copper containing food (contaminated) and food with no added copper (uncontaminated) were used. The innate immunity was measured from adult flies by determining the strength of encapsulation response. Copper concentration used in this experiment was determined based on the results of the preliminary test. The quantity of copper that had no significant effect on the mortality of the maggots or adult flies was used as a supplement of the parental larval food.

As an oviposition stimulus beef pâté was used for adults to lay eggs on. After 24 hours eggs used in the experiment were moved in covered plastic jars (parental generation) containing 50 g of beef pâté supplemented with either 200 µg copper sulfate/ g pâté (contaminated) (Sigma Aldrich copper CuSO_4_ 10.00 g for 1 liter standard solution, diluted in deionized water) or deionized water (uncontaminated). After 2 days, the maggots of both treatments were moved in the covered plastic jars, 40 maggots in each (altogether 12 plastic jars for both treatments; in total 480 maggots for both treatments), containing 45 g of either contaminated or uncontaminated food. Plastic jars were placed into large covered jars with a 2 cm layer of sawdust on the bottom for pupation. Pupae were placed separately to plastic jars daily. Hatched adults were moved to plastic boxes (21×21×15 cm) and were fed with powder food (see above) and water *ad libitum*. At the age of two days, encapsulation response and dry body mass was measured from half of the adults (copper contaminated and uncontaminated) (see below). The other half from both treatments was left for expanding the next generation.

### Experiment 2

To study whether exposure to copper during early parental development has any transgenerational effects on encapsulation response or adult mass, eggs for the next generation were collected from the parental generation at the age of two weeks (see above). The following stage of the experiment was otherwise conducted similarly to that of the parental generation (see above) but individuals from both treatments (copper contaminated and uncontaminated) were reared in food that contained no added copper. Therefore, the offspring of both treatments grew in an environment with no exposure to copper but where the amount of deionized water was the same. Otherwise the maintenance of the second generation was similar to the previous. Encapsulation response and dry body mass were measured from adult flies to study the transgenerational effects of copper on immunity and adult size (see below). During the experiment, both generations of maggots and adult flies were kept at a constant temperature of 26±1°C, at constant light.

### Parameters of encapsulation response, adult size and development time

Encapsulation response was measured from adult flies at the age of two days, by placing a 2-mm long nylon monofilament (ø 0.18, rubbed with a sand paper) into the left side of the thorax through a puncture pierced by a sterile needle. Flies were anesthetized with CO_2_ for implantation. The immune system was allowed to react to a foreign antigen for 4 hours (in the preliminary test there was found the largest individual variation) while the flies were kept individually in plastic jars. After 4 hours, implants were removed and frozen for later analyses. The strength of the encapsulation response was measured from pictures taken under a light microscope aided digital camera from two different sides [45]. The average gray value of reflected light was analyzed from pictures by using the Image J program (Image J 1.42, National Institute of Mental Health, Besheda, MD, USA). Received values were scaled so that the darkest gray value corresponded to the highest encapsulation response [45]. In previous studies the repeatability of this method has been found to be high [45], [46]. Furthermore, this method has been shown to be a biologically adequate technique. Encapsulation response against foreign particles has been found to be associated with the ability to encapsulate parasites [47], [48].

Flies were dried 24 hours at 60°C. The dry body mass of each fly was measured to the nearest 0.1 mg. Development time was counted from oviposition to adult eclosion. Emerged adults were checked once a day.

### Statistical analysis

The Kolmogorov-Smirnov test was used to test for normality. For those variables that were not normally distributed the square root transformation was used to improve normality. Where case variables were not successfully transformed, a non-parametric Mann-Whitney U test was used for analyzing the differences between treatment groups. Levene's test was used to test equality of variances. In the case of those variables that differed in equality of variances, the mean values of each vial were calculated and Independent samples t test was used in analyses to compare the differences between treatment groups. Otherwise, ANOVA or Nested ANOVA was used to compare the treatment groups. All analyses were conducted by using PASW Statistics version 18 (for Windows).
